# Targeting RNA helicase DDX3 in stem cell maintenance and teratoma formation

**DOI:** 10.18632/genesandcancer.187

**Published:** 2019-02

**Authors:** Candace L. Kerr, Guus M. Bol, Farhad Vesuna, Venu Raman

**Affiliations:** ^1^ Department of Gynecology and Obstetrics, Johns Hopkins University School of Medicine, Baltimore, MD, USA; ^2^ Department of Radiology and Radiological Science, Johns Hopkins University School of Medicine, Baltimore, MD, USA; ^3^ Department of Oncology, University Medical Center Utrecht Cancer Center, GA Utrecht, The Netherlands; ^4^ Department of Oncology, Johns Hopkins University School of Medicine, Baltimore, MD, USA; ^5^ Department of Pathology, University Medical Center Utrecht Cancer Center, GA Utrecht, The Netherlands

**Keywords:** DDX3, stem cells, differentiation, RK-33, teratoma

## Abstract

DDX3 is an RNA helicase that has antiapoptotic properties, and promotes proliferation and transformation. Besides the role of DDX3 in transformed cells, there is evidence to indicate that DDX3 expression is at its highest levels during early embryonic development and is also expressed in germ cells of adults. Even though there is a distinct pattern of DDX3 expression during embryonic development and in adults, very little is known regarding its role in embryonic stem cells and pluripotency. In this work, we examined the relationship between DDX3 and human embryonic stem cells and its differentiated lineages. DDX3 expression was analyzed by immunohistochemistry in human embryonic stem cells and embryonal carcinoma cells. From the data obtained, it was evident that DDX3 was overexpressed in undifferentiated stem cells compared to differentiated cells. Moreover, when DDX3 expression was abrogated in multiple stem cells, proliferation was decreased, but differentiation was facilitated. Importantly, this resulted in reduced potency to induce teratoma formation. Taken together, these findings indicate a distinct role for DDX3 in stem cell maintenance.

## INTRODUCTION

Despite the enormous potential of pluripotent stem cells for the treatment of human disease, a significant gap exists with respect to our understanding of the biological controls that regulate their differentiation and pluripotent nature. Thus, defining molecular markers that are essential for the maintenance of the undifferentiated state of these cells will help us understand how to drive their differentiation into more specialized cells. The discovery of factors that regulate stem cell maintenance began with the characterization of transcription factors that were required for stem cell self-renewal and pluripotency. These factors include Oct4 (also known as POU5F1 (POU domain, class 5, transcription factor 1), SRY (sex determining region Y)-box 2 (Sox2), and Nanog as well as others, which are master regulators of stem cell fate [1–4]. Since their discoveries it has been realized that mechanisms must be in place for a stem cell to maintain its pluripotent state. These mechanisms have included key players in epigenetic regulation, cell cycle, and autophagy [5–12].

Even though significant advancement has been made to understand the mechanics of stem cell maintenance and differentiation, there are still many regulatory pathways, that are not clearly understood. In our quest to categorize biomarkers for stem cell characteristics, we identified DEAD-Box Helicase 3 (DDX3) as a potential gene candidate. DDX3, also known as DDX3X because of its location on the X chromosome, is a member of the DEAD-box RNA helicase family which is involved in transcription, RNA splicing, nuclear export of mRNA, and translation initiation [13–16]. DDX proteins are expressed in a wide variety of cell types and are highly expressed during embryogenesis and in diverse human cancers. The precise roles of DDX3 in development and cancer are unclear [17, 18]. Evidence in lower organisms has indicated roles of DDX3 in early stem cell proliferation of the embryo and in mammals, regulating apoptosis and the cell cycle. In humans, the sex-specific DEAD box gene, DDX3X (also known as DBX and CAP-Rf), resides at Xp11.3-p11.23 and does not undergo X-inactivation in females [19, 20]. It has a functional homolog on the Y chromosome, DBY (91.7% homology), and this gene product has an activity that is crucial for proliferation in the spermatogonia of mice with inferred evidence in azoospermic men [21–26].

Although, a role for DDX3 has not been reported in human stem cells, its role in stem cell maintenance and differentiation is beginning to be unraveled in lower organisms. For example, the homolog of DDX3 in the planarian flatworm, *SpolvlgA*, has been demonstrated to be expressed from the first cleavage rounds in blastomere cells and blastomere-derived embryonic cells [27]. These cells are undifferentiated and they engage in a massive wave of differentiation during subsequent stage development. Interestingly, in adult worms, *SpolvlgA* is expressed in spermatogonia, spermatocytes and differentiating spermatids. Similarly in other invertebrates, the DDX3 homologue in *Botryllus schlosseri* (Urochordata), BS-PL10 modulates this animal's blastogenic cycle, increasing from blastogenic stage A to blastogenic stage D [28] while a sharp decrease in BS-PL10 expression occurs during organogenesis such that the highest levels of expression is observed in multipotent soma and germ cells. Also, Ddx3x heterozygous female mice exhibits placental abnormalities during development and is embryonic lethal [29]. In addition, loss of ddx3x results in widespread apoptosis due to enhanced DNA damage and cell cycle arrest [29]. Thus, together with the evolutionary conservation of DDX3 [30], evidence points to this as an ancestral gene with defined functional roles both in self-renewal and pluripotency.

Here, we report that DDX3 promotes stem cell maintenance. Specifically, we show that undifferentiated embryonic stem cells (ESC) and embryonal carcinoma cells (ECCs) express high levels of DDX3 compared to differentiated cells. Notably, when DDX3 activities were perturbed, we observed a drastic decrease in the proliferation of undifferentiated stem cells along with an increase in cellular differentiation. Moreover, we also confirmed *in vivo* that inhibiting DDX3 activity prevents teratoma formation in NOD-scidIL-2Rγnull (NOG) mice. Taken together, our results indicate that DDX3 is an integral component of stem cell character and regulating DDX3 activity could be used to control differentiation and pluripotency.

## RESULTS

### DDX3 expression decreases with differentiation in human ESCs and ECCs

**Figure 1:**
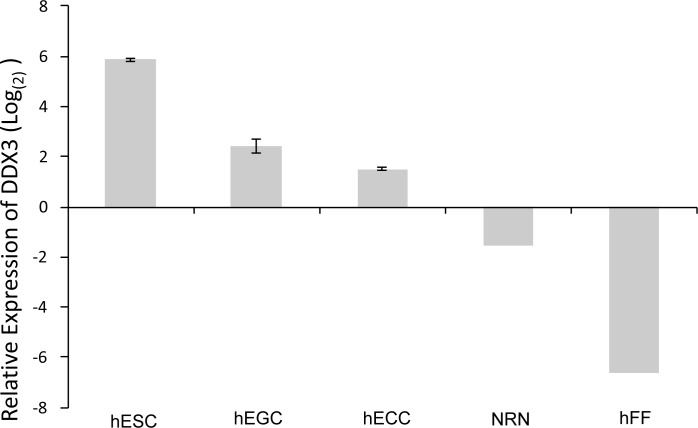
Expression of DDX3 in pluripotent and differentiated cell lines DDX3 expression is lower in differentiated cells (FF: human fetal fibroblasts; ECC Neuro: Neural differentiated hECCs) and higher in pluripotent stem cells (hEGCs, hECCs and hESCs). Relative expression of *DDX3* was compared to β-actin as the endogenous control. ΔΔCt method was also employed using the unipotent germ cell progenitor cells, PGCs as the baseline value (*N* = 3, *P* < 0.05).

Following gene expression analysis of pluripotent ESCs and unipotent progenitors of embryonic germ cells (EGCs) and ECCs known as primordial germ cells (PGCs), DDX3 was identified as one of a few genes that showed differential expression between these two cell types. To confirm this finding, qRT-PCR analysis was performed, which showed that DDX3 mRNA expression is significantly higher in ESCs and ECCs than in their differentiated counterparts of neural lineage (NRN) and human fetal fibroblasts (hFF) compared to primordial germ cells (baseline), which are the unipotent, or more differentiated progenitors of EGCs and ECCs (Figure 1). This was further corroborated by using three independent DDX3 specific primer sets (data not shown). Importantly, evidence comparing EGC to the PGC from which they are derived indicates that DDX3 may be involved in the initial stages driving pluripotency.

### Altered DDX3 expression levels following differentiation of ESCs and ECCs

**Figure 2:**
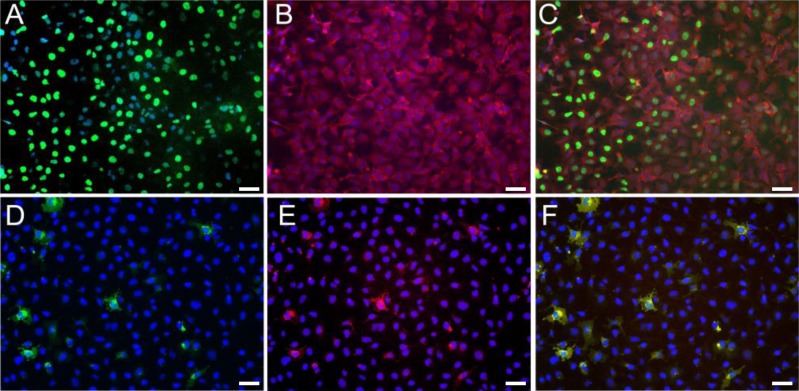
Immunofluorescence detection of DDX3 in undifferentiated human ECCs **A.** Oct4 (green), **B.** DDX3 (red). **C.** A and B overlaid. Reduced DDX3 in differentiated ECCs showing reduced expression of the pluripotent marker. **D.** Cell surface expression of Tra-1-60 (green) and **E.** DDX3 (Red) is reduced in ECCs cultured under neural inducing differentiation. **F.** Overlay of D and E shows a few remaining undifferentiated ECCs that are TRA-1-60+/DDX3+. DAPI was used as nuclear stain (blue).

As DDX3 levels were altered following differentiation, we analyzed DDX3 expression by immunofluorescence to determine the expression pattern at the cellular level. As show in Figure 2, DDX3 expression was significantly reduced after differentiation of ECCs demonstrating that undifferentiated ECCs that express OCT4 (Figure 2A) also express DDX3 (Figure 2B). More importantly, when cultured under neural-inducing conditions DDX3 expression is ablated (Figure 2E). This is evident by the lack of DDX3 expression in cells (Figure 2E) which have little or no expression of the pluripotent cell surface marker TRA-1-60 (Figure 2D) compared to the undifferentiated ECCs known to express both TRA-1-60 and OCT4. These results indicate that DDX3 expression is concomitant with pluripotent markers, especially OCT4 and TRA-1-60 expression in ECCs. Similar results were also seen in EGCs and ESCs (data not shown).

### Inhibition of DDX3 in undifferentiated hESCs reduces NANOG, OCT4 and SOX2 expression without reducing cell viability

**Figure 3:**
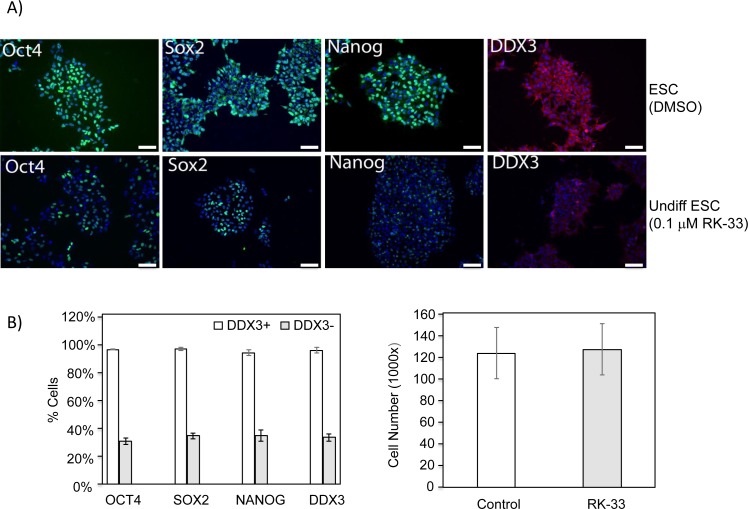
DDX3 expression correlates with pluripotency markers in hESC **A.** (Left) hESCs express DDX3 in cells expressing the stem cell markers OCT4, NANOG, and SOX2. (Right) Knock-down of DDX3 activity by RK-33 reduces the expression of NANOG, OCT4 and SOX2 in hESCs. Nucleus was stained with DAPI (Blue). **B.** Quantitative analysis of the percent of cells, which expressed each marker are presented. While the majority or >95% of the undifferentiated hESC expressed all four markers. After inhibition of DDX3 activity, the percent of cells expressing DDX3 was significantly reduced and to a similar extent as the stem cell markers. (*P* < 0.05). **C.** Bar graph of cell number with DDX3 inhibitor versus control demonstrating survival appeared unaffected by DDX3 inhibition.

Next, we carried out immunofluorescence studies on hESCs to measure the levels of protein expression of DDX3, NANOG, OCT4 and SOX2 with and without the DDX3 inhibitor, RK-33. As shown in Figure 3A, DDX3 expression was robust in undifferentiated hESCs along with the expression of NANOG, OCT4 and SOX2. It appeared also that the majority of cells, which were OCT4+ were also DDX3+. However, the addition of 0.1 μM of RK-33, a small molecule inhibitor of DDX3 [31–33], to these cells significantly decreased DDX3 levels within four days in addition to NANOG, OCT4 and SOX2 suggesting a biological function for DDX3 in maintaining the pluripotent state. Importantly, colony numbers and size was similar with and without RK-33 demonstrating that DDX3 inhibition did not affect cell viability and that RK-33 was non-toxic to the cells at the low concentration tested (Figure 3B and 3C).

### DDX3 inhibition promotes hESC differentiation

**Figure 4:**
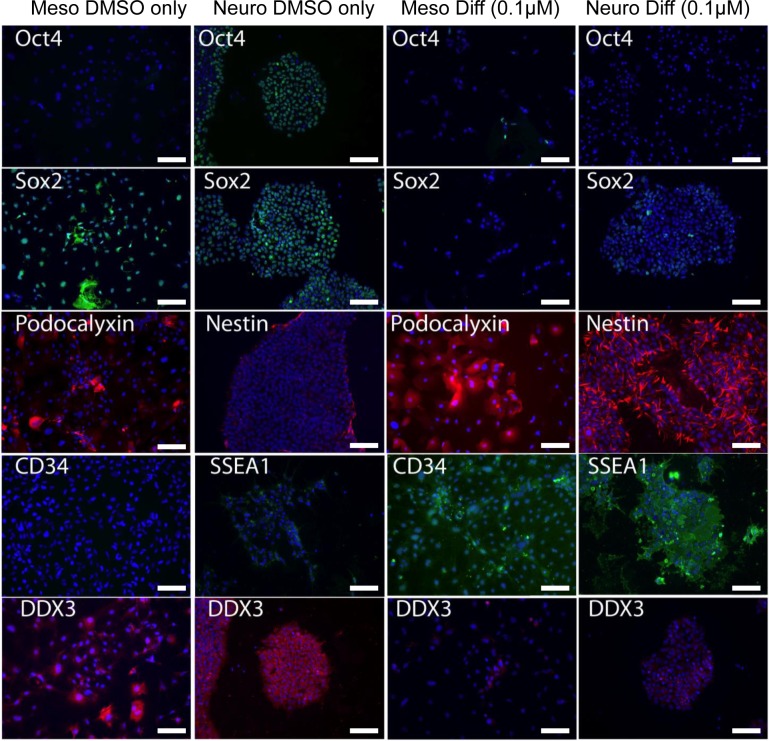
DDX3 inhibition promotes hESC differentiation after 4 days in culture Left Column vehicle only controls (DMSO). Right Column with 0.1uM RK-33. hESCs exposed to DMSO consisted of more undifferentiated OCT4+, SOX2+ cells and reduced numbers of cells expressing differentiation markers **A.** mesoderm: Podo, CD34 **B.** neuroectoderm: Nestin+, SSEA1+ compared to hESC cultured with DDX3 inhibitor RK-33. DAPI (Blue) is nuclear stain.

As down-regulating DDX3 levels reduced pluripotent markers, we wanted to determine if inhibiting DDX3 promotes differentiating phenotypes. To study this, we compared the ability of hESC to differentiate into neural and mesodermal lineages with and without DDX3 inhibition. This would confirm whether hESC differentiate more efficiently when DDX3 activity was inhibited. For this purpose, hESCs were plated onto matrigel and cultured in media that either induced neural differentiation (NEURO) or mesoderm (MESO) differentiation as previously reported by us and others [34–36]. To this media was added 0.1uM RK-33 or equivalent DMSO alone as a control and cells examined for differentiation markers after 4 days. At this early time point in differentiation, we would not expect to see a high percentage of cells that have lineage restricted markers. As shown in Figure 4, inhibition of DDX3 by RK-33 (0.1 μM) facilitated a higher number of cells with lineage restricted marker expression for both the neural and mesodermal lineage compared to controls (DMSO versus RK-33). Specifically, RK-33 reduced the number of OCT4+, SOX2+ cells and increased the number of cells which expressed neuroectoderm (SSEA1+, NESTIN+) and mesodermal (Podocalyxin+, CD34+) lineages compared to controls. Together, these results show that DDX3 is an integral effector for maintaining pluripotency.

### RK-33 does not inhibit proliferation of human embryonic fibroblast cells at physiological levels

**Figure 5:**
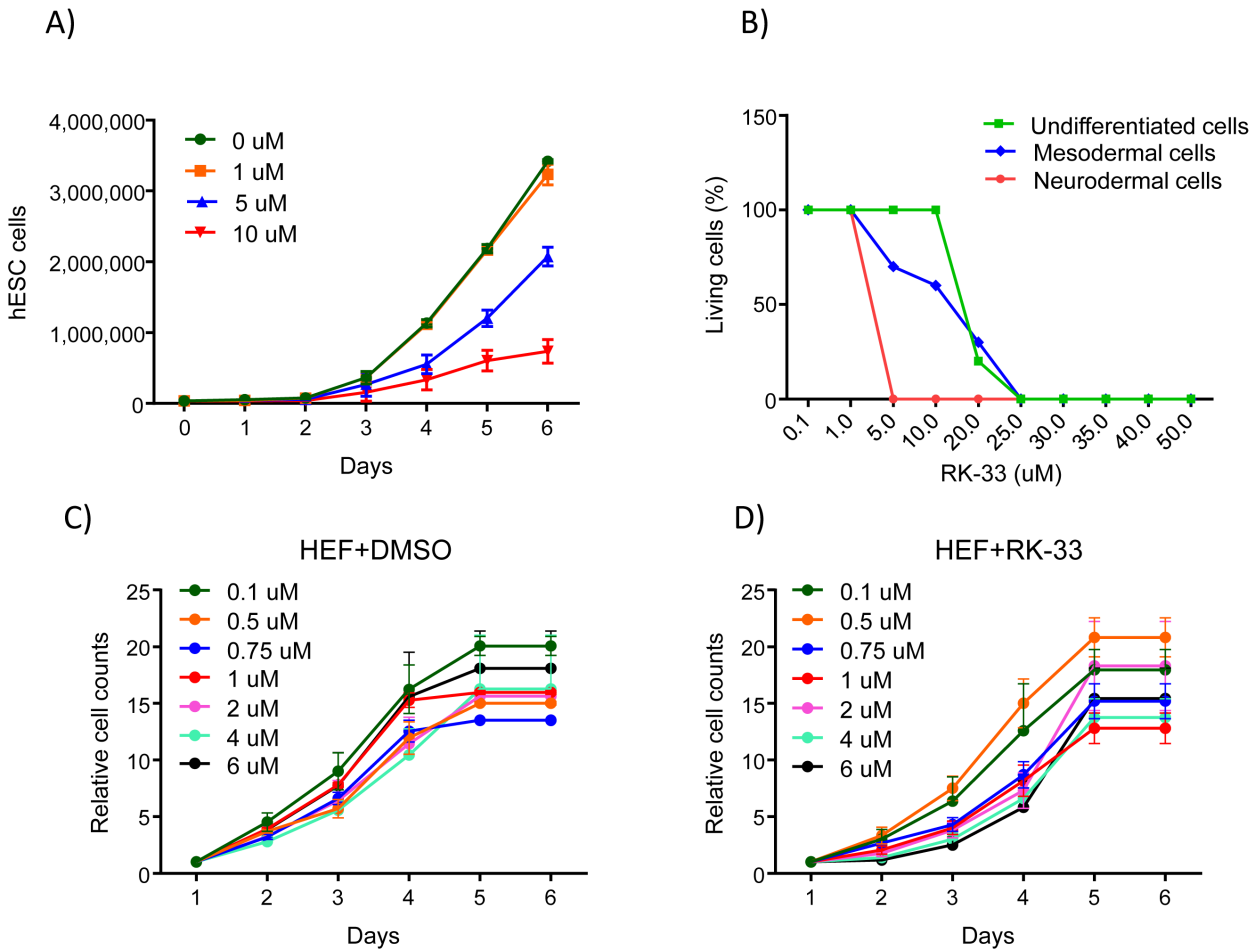
Dose response of the DDX3 inhibitor RK-33 on undifferentiated and differentiated hESCs **A.** Proliferation assay using hESCs in the presence of different concentrations of RK-33 (*n* = 2). **B.** Effect of RK-33 on undifferentiated and differentiated hESCs determined by MTT assays (*n* = 2). **C.** Effect of RK-33 on human embryonic fibroblast determined by cell counts (*n* = 2).

Given that down regulation of DDX3 by RK-33 promotes hESC differentiation, we wanted to determine whether RK-33 inhibits proliferation of the differentiated hESCs at the concentrations we studied as well as determine toxic levels of the drug. To do this we performed a dose response experiment in ESCs exposed to various concentrations of RK-33. As shown in Figure 5A, RK-33 concentration up to 1μM had no effect on hESC proliferation while 5 μM and 10 μM inhibited hESC proliferation. Moreover at higher levels, 5 μM and 10 μM, differentiated neural cells were most sensitive to RK-33 with higher reductions in living cells (0% at 5 μM of neural cells compared to mesodermal and undifferentiated cells at 0% live cells by 25 μM RK-33) (Figure 5B). Interestingly, as shown in Figure 5C and 5D, concentrations of RK-33 as used in the following studies had very little effect on the proliferation or survival of human fetal fibroblast cells further suggesting that inhibiting DDX3 is not affecting the proliferation or survival of stem cells. Taken together, these observations help support a role for DDX3 in the differentiation process.

### Reduced teratoma formation following RK-33 treatment

**Figure 6:**
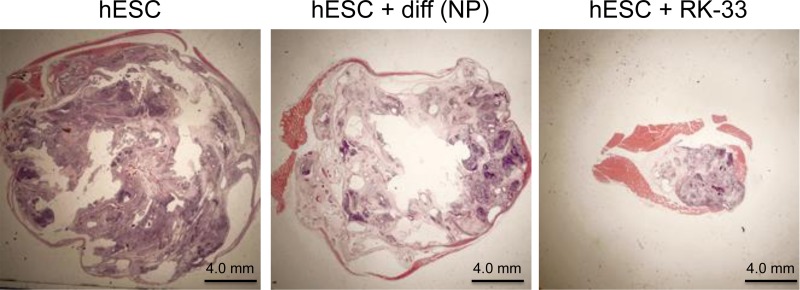
RK-33 enhances differentiation and reduces teratoma formation Five million of the respective cells in matrigel were injected into the flanks of NOG mice. Following 8 weeks of incubation, tumor volumes were measured and the samples fixed, sectioned and stained with H&E. The pictures shown represent the actual teratoma volume as related to pixel ratio. The pictures were taken using a 1X lens. Four animals per group were used for this study.

Based on our earlier observation that RK-33 facilitates differentiation and decreases pluripotency markers, we carried out a pilot experiment to determine if hESCs treated with RK-33 can reduce teratoma formation. As shown in Figure 6, hESCs when injected into the flanks of NOG mice resulted in an average teratoma volume of over 2000mm^3^. However, the teratoma volume from hESCs animals treated with RK-33 was barely palpable (*N* = 12 injections with or without RK-33 treatment). This indicates that inhibiting DDX3 activity can enhance differentiation and decrease teratoma growth.

## DISCUSSION

A role for DDX3 in stem cell maintenance and differentiation is beginning to be unraveled in lower organisms. For example, the homologue of DDX3 in the planarian flatworm, SpolvlgA, are expressed from the first cleavage rounds in blastomere cells and blastomere-derived embryonic cells [27]. These cells are undifferentiated and they engage in a massive wave of differentiation during subsequent stage development. Interestingly, in adult worms, SpolvlgA is expressed in spermatogonia, spermatocytes and differentiating spermatids. Similarly in other invertebrates, the DDX3 homologue in Botryllus schlosseri (Urochordata), BS-PL10 modulates this animal's blastogenic cycle, increasing from blastogenic stage A to blastogenic stage D [28] while, a sharp decrease in BS-PL10 expression occurred during organogenesis such that the highest levels of expression occurred in multipotent soma and germ cells. Thus, together with the evolutionary conservation of DDX3 [30], evidence points to this as an ancestral gene with defined functional roles both in differentiation and pluripotency. In addition, DDX3 has been associated with the less differentiated phenotypes of some cancers [31–33, 37–39]. For example, we have found that DDX3 activity has been associated with the proliferation and differentiated status of breast cancer cells [38, 40].

We now show that DDX3 has a role in differentiation by showing its involvement in the pluripotent state of stem cells. Pluripotent stem cells are defined by their clonal capacity and ability to differentiation into all cell types of the embryo. While they share certain features with cancer cells such as high proliferative capacity and immortality in vitro, they also exhibit a robust differentiation capacity that provides a unique opportunity to study the role of DDX3 in proliferation and their differentiation, which closely mimics development. Using these models, we were able to compare more differentiated cells such as primordial germ cells (PGCs), the progenitor germ cells for sperm and egg, to their undifferentiated pluripotent counterparts, embryonic germ cells (EGCs) and embryonal carcinoma cells (ECCs). EGCs and ECCs are both pluripotent stem cells derived from PGCs, EGCs are derived from PGCs in cell culture while ECCs are cancer stem cells derived from teratocarcinomas organically derived from PGCs in the body. Here, we show that compared to a known differentiated progenitor cell, PGCs DDX3 expression was higher in EGCs and ECCs. DDX3 expression was also higher in ECCs and embryonic stem cells (ESCs) compared to their differentiated progeny. ESCs are pluripotent stem cells derived from embryonic blastocyst that like EGCs are unique from ECCs as their proliferation is controlled by cell-to-cell contact and they are genomically stable. In addition to its effect on ECCs and EGCs (data not shown), we found that DDX3 selectively affected proliferation of undifferentiated ESCs, while proliferation of differentiated cells appeared unaltered. This indicates that DDX3 plays a key role in pluripotent stem cell proliferation and its maintenance of the undifferentiated state. Moreover, we found that DDX3 inhibition facilitates differentiation in ECCs and ESCs, and represses pluripotency factors. As a result, inhibition of DDX3 in vivo prevented teratoma formation in NOG mice.

The role in cell cycle regulation and the undifferentiated state of many different types of human pluripotent stem cells indicates that DDX3 has the potential to be a key component of mechanisms that are essential for the maintenance of stem cell renewal and pluripotency. In fact, given the broad functions DDX3 performs across various cell types and with evidence that it effects protein translation in inverse directions depending on the system (5, 26), we anticipate that DDX3 probably works in concert with several factors to produce its effects on the stem cell phenotype. For this reason, DDX3 maybe a promising component to target for regenerative medicine. Thus, it would also be of interest in future studies to determine whether DDX3 could also be exploited to generate patient-specific iPSCs for disease modeling, drug testing, or regenerative medicine with patient-derived cells.

## MATERIALS AND METHODS

### Ethics statement

All animal experiments were conducted in accordance with a protocol approved by the Johns Hopkins University Animal Care and Use Committee (Protocol# MO11M279). All mice were housed in a sterile environment where they had free access to food and water as outlined in our institutional guidelines. Human tissue was obtained as previously described from the University of Washington's NIH-supported tissue repository with Johns Hopkins University Institutional Review Board approval.

### Cell culture

Human ESCs from the H1 line (WiCell, Fed ID# 0043), human fetal fibroblasts (ATCC, HFF-1) human primordial germ cells (PGCs) and human embryonic germ cells (EGCs) derived from PGCs by the Kerr laboratory as described [41] were cultured on matrigel (BD Biosciences) in 10 cm cell culture dishes with Dulbecco's modified Eagle's medium/F12-Knockout serum-based media conditioned by mouse embryonic fibroblast cells (MEFs) and supplemented with 4ng/mL fibroblast growth factor 2 (FGF2) as described previously [42]. ESCs were routinely maintained on MEFs mitotically inactivated with 5,000 rads of γ-radiation and passaged every 3-5 days after disaggregation with 1 mg/ml collagenase in D-PBS. The human embryonal carcinoma line, NTERA-2 cl.D1 (ATCC) was cultured on matrigel-coated plates in 15% FCS supplemented DMEM media as previously described [42, 43].

### Immunocytochemistry and quantitative analysis

For immunocytochemistry, cells were fixed in 4%PFA for 10 minutes followed by 10 minutes in 0.3% triton in PBS. All antibodies except DDX3, which was made in our laboratory [31–33, 37, 38] is commercially available. Briefly, antibodies were diluted in 15% goat serum in DPBS (1:50 dilution) and incubated with tissue for 1 hr at room temperature. All antibodies were detected using fluorescently labeled secondary antibodies (1:200 dilution; Millipore) in 15% goat serum in DPBS for 1 hr at room temperature. Cells were counterstained with DAPI (Sigma) to detect nuclei. Negative controls were performed using secondary antibodies alone. For fluorescent quantitative analysis, images were captured using a Nikon E800 microscope and imported to Metamorph Imaging Software (Version 7.7). Total fluorescence units (FU) were calculated by dividing the number of pixels per area (3 areas measured per image), which are in arbitrary units. An adjacent area of the field that was not of interest was also measured to establish background fluorescence as previously described [44] At least, three independent experiments were performed for each treatment from which three separate 48-count tissue culture wells were counted per stain.

### MTT cell proliferation assays

Cells (5,000) were plated onto 96 well plates coated in matrigel with conditioned media obtained from mouse embryonic fibroblasts as described [45, 46]. The media was replaced daily. MTT assays (Invitrogen) were performed daily for 5 days using 100 μl of MTT solution (5 mg/ml) added to each well and incubated for 3 h at 37°C according to manufacturer's instructions. The formed MTT formazan crystals were dissolved with 500 μL DMSO, and the spectrophotometric assay was carried out at 590 nm as described. Each condition was done in quadruplicate, and 2 independent experiments were performed.

### Teratoma assay

Five million undifferentiated ESCs were injected subcutaneously into 12 NOD-scidIL-2Rγnull (NOG) mice as previously described [47]. 24 hours after ESCs transplantation, mice were injected IP with 20mg/Kg RK-33 or DMSO as control and then repeated injected every other day until sacrifice. After 8 weeks, teratomas were fixed in 4% PFA, embedded in paraffin at 60°C and then cut into 10 micron sections and stained with hematoxylin-eosin.

### Statistical analysis

Expression levels of DDX3 and the other proteins were compared by chi-square test or t-test as indicated. Logistic regression or ANOVA was used for multivariate analysis. Pearson correlation coefficient was determined for correlation analysis.

All statistical analyses were carried out with SPSS 17.0 for Windows. (SPSS Inc., Chicago, IL, USA), regarding two-sided p-values below 0.05 as significant.

## Abbreviations

ECCs: embryonal carcinoma cells
EGCs: embryonic germ cells
ESCs: embryonic stem cells
FGF2: fibroblast growth factor 2
FU: fluorescence units
hFF: human fetal fibroblasts
MEFs: mouse embryonic fibroblast cells
MTT: tetrazolium MTT (3-(4, 5-dimethylthiazolyl-2)-2,5-diphenyltetrazolium bromide)
NOG: NOD/Shi-scid/IL-2Rγ^null^
NRN: neural lineage
PGCs: primordial germ cells
